# Thrombin-activated interleukin-1α drives atherogenesis, but also promotes vascular smooth muscle cell proliferation and collagen production

**DOI:** 10.1093/cvr/cvad091

**Published:** 2023-06-13

**Authors:** Laura C Burzynski, Alejandra Morales-Maldonado, Amanda Rodgers, Lauren A Kitt, Melanie Humphry, Nichola Figg, Martin R Bennett, Murray C H Clarke

**Affiliations:** Section of CardioRespiratory Medicine, The Heart and Lung Research Institute, The University of Cambridge, Papworth Road, Cambridge Biomedical Campus, Cambridge CB2 0BB, UK; Section of CardioRespiratory Medicine, The Heart and Lung Research Institute, The University of Cambridge, Papworth Road, Cambridge Biomedical Campus, Cambridge CB2 0BB, UK; Section of CardioRespiratory Medicine, The Heart and Lung Research Institute, The University of Cambridge, Papworth Road, Cambridge Biomedical Campus, Cambridge CB2 0BB, UK; Section of CardioRespiratory Medicine, The Heart and Lung Research Institute, The University of Cambridge, Papworth Road, Cambridge Biomedical Campus, Cambridge CB2 0BB, UK; Section of CardioRespiratory Medicine, The Heart and Lung Research Institute, The University of Cambridge, Papworth Road, Cambridge Biomedical Campus, Cambridge CB2 0BB, UK; Section of CardioRespiratory Medicine, The Heart and Lung Research Institute, The University of Cambridge, Papworth Road, Cambridge Biomedical Campus, Cambridge CB2 0BB, UK; Section of CardioRespiratory Medicine, The Heart and Lung Research Institute, The University of Cambridge, Papworth Road, Cambridge Biomedical Campus, Cambridge CB2 0BB, UK; Section of CardioRespiratory Medicine, The Heart and Lung Research Institute, The University of Cambridge, Papworth Road, Cambridge Biomedical Campus, Cambridge CB2 0BB, UK

**Keywords:** Atherosclerosis, Inflammation, Coagulation, IL-1, Thrombin

## Abstract

**Aims:**

Atherosclerosis is driven by multiple processes across multiple body systems. For example, the innate immune system drives both atherogenesis and plaque rupture via inflammation, while coronary artery-occluding thrombi formed by the coagulation system cause myocardial infarction and death. However, the interplay between these systems during atherogenesis is understudied. We recently showed that coagulation and immunity are fundamentally linked by the activation of interleukin-1α (IL-1α) by thrombin, and generated a novel knock-in mouse in which thrombin cannot activate endogenous IL-1α [IL-1α thrombin mutant (IL-1αTM)].

**Methods and results:**

Here, we show significantly reduced atherosclerotic plaque formation in IL-1αTM/*Apoe*^−/−^ mice compared with *Apoe*^−/−^ and reduced T-cell infiltration. However, IL-1αTM/*Apoe*^−/−^ plaques have reduced vascular smooth muscle cells, collagen, and fibrous caps, indicative of a more unstable phenotype. Interestingly, the reduced atherogenesis seen with thrombin inhibition was absent in IL-1αTM/*Apoe*^−/−^ mice, suggesting that thrombin inhibitors can affect atherosclerosis via reduced IL-1α activation. Finally, bone marrow chimeras show that thrombin-activated IL-1α is derived from both vessel wall and myeloid cells.

**Conclusions:**

Together, we reveal that the atherogenic effect of ongoing coagulation is, in part, mediated via thrombin cleavage of IL-1α. This not only highlights the importance of interplay between systems during disease and the potential for therapeutically targeting IL-1α and/or thrombin, but also forewarns that IL-1 may have a role in plaque stabilization.

**Time of primary review: 33 days**

## 1. Introduction

Coagulation and inflammation are intrinsically linked. Indeed, the coagulation system likely developed from an early innate immune system, with blood serine proteases diverging from complement proteases.^1^ In mammals, inflammation can induce expression of tissue factor to promote coagulation,^2^ while thrombin cleavage of protease-activated receptors (PARs) induces cytokine and adhesion molecule expression, leading to inflammation.^2^ Coagulation is rapid, with the intrinsic or extrinsic pathways triggering a protease cascade that activates thrombin, leading to fibrin deposition, platelet activation and haemostasis. Innate immunity is slower and typically requires sensing of pathogen-associated molecular patterns via cognate receptors to induce expression of cytokines that direct inflammation and subsequent adaptive immunity.^3^ Importantly, we have recently identified a direct link between haemostasis and immunity in mammals, whereby thrombin cleaves and activates interleukin-1α (IL-1α),^4^ resulting in direct and rapid induction of inflammation.

The potential for thrombin activation of IL-1α is particularly interesting in the context of atherosclerosis and other vascular diseases. Although plaque rupture causes acute thrombosis and vessel occlusion, leading to myocardial infarction (MI) or stroke, strong evidence suggests that chronic ongoing coagulation drives plaque growth. For example, human plaques without fissures or ulceration contain large amounts of fibrin that is localized throughout,^5^ while early plaques contain clotting factors and thrombin:antithrombin (TAT) complexes.^6^ Indeed, levels of TAT complexes predict the severity of coronary atherosclerosis.^7^ Furthermore, inducing a hypercoagulable state in ApoE mice by knocking out Heparin Cofactor II^8^ or tissue factor pathway inhibitor^9^ increases plaque size. Conversely, thrombin inhibition reduces and stabilizes plaques.^10,11^ However, knockout of PAR4 has no effect on plaques in ApoE mice,^12^ while PAR2 loss only modestly reduces athero,^13^ suggesting that the action of thrombin is not all mediated via PARs. Together, this implies that coagulation and thrombin activation occurs chronically throughout atherogenesis, not just at acute plaque rupture, and that this drives plaque growth. IL-1α is expressed by vascular smooth muscle cells (VSMCs), endothelial cells (ECs), and macrophages, and is released upon necrosis or during inflammasome activation. However, whether thrombin-activated IL-1α contributes to atherogenesis is currently not known.

IL-1 signalling is now established to be causative of the clinical manifestations of atherosclerosis, with CANTOS showing significantly reduced major adverse cardiovascular events (MACEs) and mortality in patients treated with the IL-1β inhibitor canakinumab.^14,15^ IL-1α and IL-1β bind the Type 1 IL-1 receptor (IL-1R1) and induce identical proinflammatory effects,^3^ including cytokine secretion, upregulation of adhesion, MHC and costimulatory molecules, and vascular leakage.^16^ IL-1 also has powerful effects on adaptive immunity by enhancing survival and expansion of T cells, T_H_17-cell differentiation, and effector T-cell proliferation with Tregs present^17^—all effects important during atherogenesis. Both IL-1α and IL-1β are synthesized as proforms that require cleavage for full biological activity. IL-1α is also an important damage-associated molecular pattern released upon necrosis,^18–24^ but cytokine activity is controlled in a cell type–dependent manner.^25^ Multiple studies find that IL-1 signalling drives plaque growth,^26–36^ suggesting a consensus for a proatherogenic role of IL-1. However, controversies exist; for example, while reduced atherosclerosis is seen in IL-1α^−/−^ mice,^31,36,37^ lesions are either reduced^27^ or unchanged^31,36^ in IL-1β^−/−^ mice. Another study using IL-1R1^−/−^ mice found smaller plaques that were more unstable, suggesting that although IL-1 drives plaque growth, it could also have a stabilizing effect.^38^ Cholesterol crystals activate inflammasomes^33,39^ and thus IL-1β; however, deficiency in the key inflammasome factors NLRP3 or ASC showed either reduced plaque^33^ or no change.^40^ Such discrepancies are currently unexplained, but could reflect different temporal roles for IL-1α and IL-1β,^41^ and/or inflammasome-independent activation of IL-1α, such as by thrombin.^4^

Here, we present novel data revealing that a direct link between the coagulation and the immune system drives atherogenesis independent of plaque rupture or frank thrombus formation. Thus, IL-1α thrombin mutant (IL-1αTM)/*Apoe*^−/−^ mice (in which thrombin cannot activate IL-1α) have smaller atherosclerotic plaques with less T-cell infiltration and were refractory to the reduction in plaque size usually seen with thrombin inhibition. However, IL-1αTM/*Apoe*^−/−^ plaques also have reduced VSMCs, collagen, and fibrous caps, indicative of a more unstable phenotype. Indeed, IL-1α drives VSMC proliferation and collagen expression *in vitro*. Thus, although thrombin-cleaved IL-1α drives plaque growth, in keeping with the consensus for IL-1 driving atherogenesis,^26–36^ IL-1 may also play an important role in plaque stabilization.

## 2. Methods

All materials are from Sigma (St. Louis, MO) unless stated otherwise.

Animal protocols were performed under UK Home Office licensing. IL-1αTM mice were generated by homologous recombination of the R114Q point mutation into Exon 5 of *Il1a* in an FLP ES cell line using an FRT-flanked Neo selection cassette, followed by standard generation of chimaeras (inGenious Targeting Laboratory; Ronkonkoma, NY).^4^ IL-1αTM mice on a C57BL/6J background were crossed to *Apoe*^−/−^ (Apoe^tm1Unc^; Jax) to generate IL-1αTM^−/−^/*Apoe*^−/−^ and IL-1αTM^+/+^/*Apoe*^−/−^ littermates, which were born at expected frequencies with no gross phenotype. Mice were maintained on a 12 h light/dark cycle and normal chow (#105, SAFE) and water were available ad libitum. Euthanasia was via a rising concentration of CO_2_. For experimental atherosclerosis, males were fed a high fat (HF) ‘Western’ diet (#829100; SDS) from 6 weeks for 10 or 12 weeks, as indicated. Serum lipids were profiled at 6 weeks (Siemens Dimension RXL). Full blood counts used a scil Vet ABC+ (Horiba). Dabigatran (Pradaxa) was mixed at 10 g/kg of powdered HF diet.^11^ Bone marrow chimeras were generated by standard methods. Briefly, mice were irradiated with a split dose of 5.5 grey, 4 h apart, and 10 × 10^6^ bone marrow cells injected via the tail vein within 2 h of the final irradiation. Mice were left to reconstitute for 4 weeks before HF feeding. Parallel experiments with CD45.1/2 showed ∼95% donor engraftment, with normal blood counts within 4 weeks (see Supplementary material online, *Figure S8*). Briefly, whole blood was incubated with anti-CD45.1 and anti-CD45.2 (1:50, 1:80; 30 min; both BioLegend, San Diego, CA), RBCs lysed (eBioscience, Waltham, MA), washed, and re-suspended in FACS buffer and analysed by flow cytometry (Accuri C6).

### 2.1 Immunohistochemistry and morphometry

Mouse tissues were fixed in 10% formalin overnight, before processing, paraffin embedding, and 5 μm sectioning. Aortic root plaques were serial sectioned from the start of the valve leaflets, and at every 100 μm towards the heart, slides were cleared before antigen retrieval with sodium citrate (10 mM; pH 6), blocking in H_2_O_2_ (3%; 10 min), and then horse serum (5%; 1 h), before incubation with anti-CD3 (1:75; NCL-CD3-12; Novacastra, UK), anti-αSMA (1:400; 1A4; Dako, Santa Clara, CA), anti-Mac-3 (1:400; M3/84; BD, Franklin Lakes, NJ), or isotype controls (Abcam, UK; all 16 h, 4°C). Washed αSMA and Mac-3 sections were incubated with biotinylated 2ry antibody [1:500; 1 h, room temperature (RT)], then ABComplex (30 min, RT), before visualization with DAB (all Vector, Newark, CA), while CD3 used an Abcam kit (ab64264). Collagen was visualized with Masson’s trichrome staining (HT15). Imaging was performed on a BX51 (Olympus, Japan) using Image-Pro Insight 9.1 software (Media Cybernetics, Rockville, MD). Plaque area was identified by H&E; necrotic core as the acellular cholesterol cleft-rich area; fibrous cap as the VSMC and proteoglycan-rich area underlying the endothelial layer; media as the area between the internal and external elastic lamina. Plaque constituent areas were quantified as number of DAB (CD3, αSMA, Mac-3) or blue (Masson’s) positive pixels as a percentage of total plaque pixels (see Supplementary material online, *Figures S5* and *S6*). Non-specific staining of the necrotic core was excluded from analysis. For Oil red O staining, aortas were cleaned of adipose, rinsed in isopropanol (60%; 30 s), incubated with Oil red O (1.2 mg/mL in 60% isopropanol; 15 min), rinsed in isopropanol (60%; 30 s), then H_2_O (2 min), before tiled imaging using a ×10 objective.

### 2.2 Spleen and whole-blood immunoprofiling

Spleens were sieved (70 µm), before washing [phosphate-buffered saline (PBS); 350 g, 5 min], re-sieving (40 µm), RBC lysis (eBioscience), washing, and resuspension in FACS buffer (1% BSA, 0.05% NaN_3_, in PBS) or full RPMI 1640. Cells in FACS buffer were Fc blocked (1:100; BioLegend; 10 min, RT) before staining for T-cell activation with: anti-CD4 (1:800; eBioscience), anti-CD8 (1:100), anti-CD62L (1:80), anti-CD44 (1:400; all BioLegend; 20 min, RT); or for Tregs with: anti-CD4 (1:800), anti-CD25 (1:80; BioLegend; 20 min, RT), before washing, fixation, permeabilization (FOXP3 Fix/Perm; BioLegend), then anti-FOXP3 (1:20; BioLegend; 30 min, RT). Splenocytes in RPMI were treated ±ionomycin/phorbol 12-myristate 13-acetate/brefeldin A (1:500; BioLegend), incubated (5 h, 37°C), washed, resuspended in FACS buffer, Fc blocked, stained with anti-CD4 or anti-CD8 (20 min, RT), washed, fixed, permeabilized (BioLegend), then anti-IL-10, anti-IL-17, and anti-interferon γ (IFNγ; all 1:100; BioLegend; 30 min, RT). For neutrophil, monocyte or Ly6C^+^ cells, whole blood (ethylenediaminetetraacetic acid) was stained with anti-CD115 (1:100; eBioscience), anti-CD11b (1:800), anti-Ly6G (1:80; both BioLegend), anti-Ly6C (1:400; AbD Serotec, Hercules, CA), at RT for 30 min, before RBC lysis, washing, and analysis by flow cytometry (Accuri C6).

### 2.3 Cell culture

Primary murine adult ear fibroblasts and primary human aortic VSMCs (two isolates from different individuals) were cultured in DMEM, 10% fetal calf serum (FCS), 10 U/mL penicillin, 10 mg/mL streptomycin, 5 mg/mL l-glutamine, and were passaged at 80% confluence. Mouse bone marrow–derived macrophages (BMDMs) were cultured in RPMI 1640 supplemented with 2 mM l-glutamine, 100 U/mL penicillin, 10 μg/mL streptomycin, 50 μM β-mercaptoethanol, and 10% FCS, with 15% L929 conditioned media during mBMDM differentiation. Briefly, bone marrow was flushed from femurs and tibias, washed, cells plated, and media replenished every other day. Where indicated cells were treated with IL-1α (20 ng/mL; PeproTech, UK); calpeptin (30 μM; Enzo, UK); lipopolysaccharide (LPS) (1 µg/mL); SB203580 (50–500 nM); isohelenin (0.5–5 μM; Santa Crap); dimethylsulfoxide (DMSO).

### 2.4 Cleavage of macrophage-derived pro-IL-1α

Control and IL-1αTM BMDMs treated with LPS (1 μg/mL; 6 h, 37°C) were lysed in 20 mM Tris pH 8.4, 150 mM NaCl, 2.5 mM CaCl_2_ by freeze/thaw, debris removed by centrifugation and the pro-IL-1α-containing lysate incubated (2 h, RT) ±thrombin (0.09 U/mL; Novagen, UK), ±PPACK (100 µM; Enzo), with reactions stopped by addition of Laemmli buffer. Where indicated, BMDMs were pre-incubated with calpeptin before lysis (20 min, 37°C).

### 2.5 IL-1 bioassay, enzyme-linked immunosorbent assay, and thrombin assay

Murine fibroblasts were adhered overnight in full media. Media was replaced along with test treatments as indicated, and incubated for 6 h. Specific IL-1α activity was inferred with a neutralizing antibody against mouse IL-1α (2 µg/mL; R&D), added throughout the 6 h incubation. Conditioned media was collected, clarified, and mouse IL-6 assayed by bead enzyme-linked immunosorbent assay (ELISA; ThermoFisher, Waltham, MA) as per the manufacturer’s instructions. Beads were analysed by flow cytometry (Accuri C6). Serum IL-1α was measured by ELISA (DuoSet; R&D) after a 1:3 dilution, while serum TAT was measured by ELISA (Abcam) after a 1:50 dilution and absorbance measured on a plate reader (BMG LABTECH, UK). Thrombin activity was measured with a fluorogenic substrate (605211; EMD Millipore, UK). Briefly, 10 μL of diluted serum (as indicated) was mixed with 10 μL of substrate (0.25 mM final) in 80 μL of PBS, and incubated at 37°C for 20 min before measuring fluorescence.

### 2.6 Assessment of VSMC proliferation

Human aortic VSMCs were plated at ∼30% confluence in full media, allowed to adhere overnight, media replaced along with treatments as indicated and incubated for 7 days (37°C). Media and treatments were refreshed every 3 days. Proliferation was assessed by addition of Alamar Blue (1:10; 1 h, 37°C; Invitrogen, Waltham, MA) and absorbance measurement at 600 nm, followed by cell fixation (3:1 methanol:acetic acid; 5 min, RT), staining with crystal violet (0.1% in PBS; 10 min, RT), solubilization of dye with acetic acid (10%) and absorbance measurement at 600 nm.

### 2.7 Western blotting and quantitative polymerase chain reaction

Westerns were performed as previously described with lysis of cells directly in Laemmli buffer, SDS–PAGE and transfer onto polyvinylidene difluoride membrane. After blocking (5% milk) membranes were incubated (16 h, 4°C) with mouse IL-1α pAb (1:500; R&D), before washing (PBS/Tween) and incubation (1 h, RT) with anti-mouse horseradish peroxidase (1:2000; GE, Chicago, IL). After washing, membranes were visualized with ECL reagent (Amersham, UK) and X-ray film (Fujifilm, Japan). Quantitative polymerase chain reaction was performed after RNA extraction (RNeasy; Qiagen, Germany) and cDNA synthesis (RT system; Promega, Madison, WI) using SsoAdvanced SYBR green master mix (Bio-Rad, Hercules, CA) and a CFX Connect thermocycler (Bio-Rad). Relative expression was calculated by the ddCt method with *B2M* as the reference gene. Primer sequences: *COL1A1*: GATTCCCTGGACCTAAAGGTGC and AGCCTCTCCATCTTTGCCAGCA; *COL1A2*: CCTGGTGCT AAAGGAGAAAGAGG and ATCACCACGACTTCCAGCAGGA; *COL3A1*: TGGTCTGCAAGGAAT GCCTGGA and TCTTTCCCTGGGACACCATCAG; *B2M*: GAGGCTATCCAGCGTACTCCA and CGG CAGGCATACTCATCTTTT.

### 2.8 Statistics

Data are presented as mean ± standard error mean (SEM), unless otherwise stated. All statistical analyses were carried out using Prism 7 (GraphPad, Boston, MA). All assays that produced continuous data, with the exception of flow cytometry and mouse experiments, were performed in duplicate. *n* = an individual experimental replicate performed on a different day, or an individual mouse—never a technical replicate. Before statistical testing for significance, data were analysed for normality with a Shapiro–Wilks test, with normal distribution analysed by parametric and non-normal by non-parametric. Parametric test analysis of continuous data used unpaired *t*-test (two-tailed) or ANOVA with Dunnett’s *post hoc* or Tukey’s *post hoc* multiple comparisons test. Non-parametric tests used the Mann–Whitney *U* test or the Kruskal–Wallis test.

## 3. Results

### 3.1 Mutation of the thrombin site in IL-1α prevents its cleavage and activation

**Figure 1:**
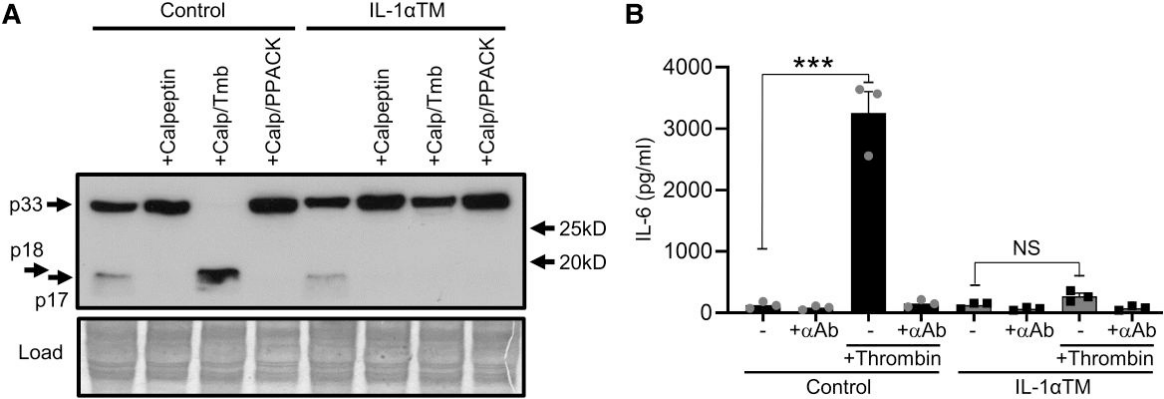
Mutation of the thrombin site in IL-1α prevents its cleavage and activation. (*A*) Western blot for IL-1α derived from LPS stimulated *Il1a*^WT/WT^ (control) and *Il1a*^R114Q/R114Q^ (IL-1αTM) macrophages treated ±calpeptin (Calp), ±thrombin (Tmb), or ±PPACK, showing cleavage of pro-IL-1α (p33) to a ∼p17 form by calpain and p18 form by thrombin in control samples, but a failure of thrombin to cleave pro-IL-1α in IL-1αTM samples. (*B*) IL-1α-dependent IL-6 production by mouse fibroblasts incubated with thrombin-cleaved control or IL-1αTM pro-IL-1α, ±a neutralizing IL-1α antibody (+αAb). Data represent mean ± SEM; *n* = 3 (*B*); representative of *n* = 2 (*A*). ***P = ≤ 0.001; NS, not significant.

We have recently shown that mammalian IL-1α contains a highly conserved consensus site that is targeted by thrombin, resulting in cleavage and activation of IL-1α.^4^ We mutated the key Arginine residue of this site to Glutamine (R^114^Q) within the endogenous mouse *Il1a* gene and bred to homozygosity (*Il1a*^R114Q/R114Q^) to produce IL-1αTM mice. Using LPS-treated BMDMs as a source of pro-IL-1α, we show that canonical cleavage of IL-1α by calpain occurs in wild-type (control) and IL-1αTM mice (*Figure 1A*; as evidenced by loss of the p17 band with calpeptin), but thrombin cannot process pro-IL-1α derived from IL-1αTM mice (*Figure 1A*). Concomitantly, thrombin treatment of pro-IL-1α from control mice induces high levels of IL-1α-specific activity (as evidenced by reduced IL-6 with an anti-IL-1α Ab), while pro-IL-1α from IL-1αTM mice does not (*Figure 1B*). No other differences in physiological parameters (full blood count or clotting parameters; spleen/lymph node weight) or typical IL-1 responses (pro-IL-1α/β expression; cell surface IL-1α; IL-1α/β release after inflammasome activation; T-cell number, subtypes, or polarization; Treg number) was found between genotypes,^4^ indicating the only tangible difference between control and IL-1αTM mice is the ability of thrombin to cleave and activate IL-1α.

### 3.2 IL-1αTM/*Apoe*^−/−^ mice generate less atherosclerotic plaque than *Apoe*^−/−^ mice

**Figure 2:**
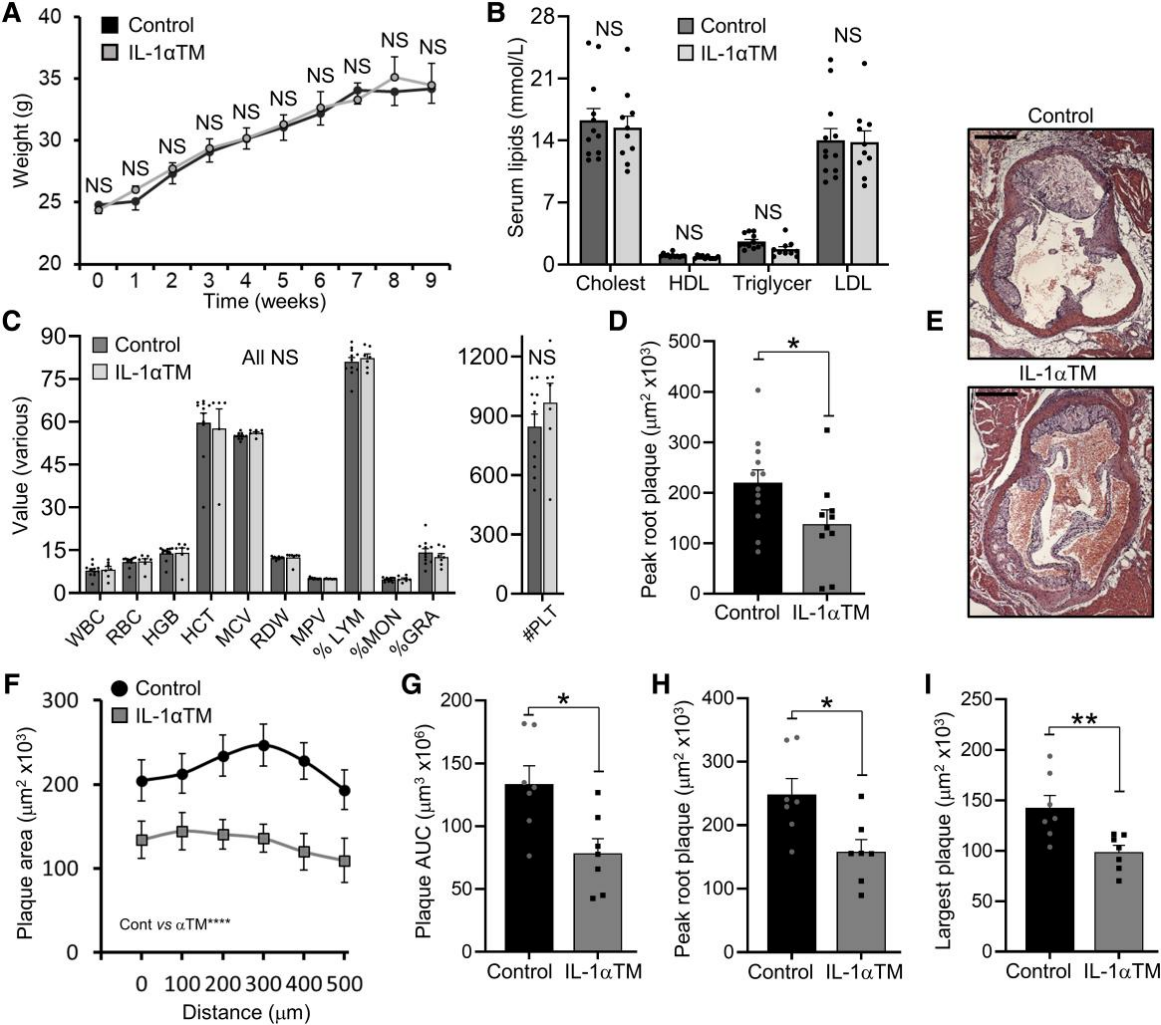
IL-1αTM/*Apoe*^−/−^ mice generate less atherosclerotic plaque than *Apoe*^−/−^ mice. *Apoe*^−/−^ (control) and IL-1αTM/*Apoe*^−/−^ (IL-1αTM) mice were fed a HF diet for 10 weeks, with total body weight measured longitudinally (*A*), and serum lipid concentrations (*B*) and full blood counts (*C*) measured at 6 weeks. Analysis of aortic root plaque in IL-1αTM/*Apoe*^−/−^ and *Apoe*^−/−^ mice by quantification of ‘traditional’ peak plaque (*D* and *E*), or aortic root plaque serial sections quantified (*F*) and analysed for AUC (*G*), peak plaque area (*H*), and single largest plaque (*I*). White/red blood cell (W/RBC); haemoglobin (HGB); haematocrit (HCT); mean corpuscular volume (MCV); RBC distribution width (RDW); mean platelet volume (MPV); lymphocyte (LYM); monocyte (MON); granulocyte (GRA); platelet count (#PLT). Data represent mean ± SEM; *n* = 12/10 (*A*, *B*, *D*), 11/7 (*C*), and 7/7 (*F–I*) (control/IL-1αTM) mice. *P = ≤ 0.05; NS = not significant. Scale bar = 500μm.

To investigate if thrombin-cleaved IL-1α alters atherogenesis, we crossed IL-1αTM mice with *Apoe*^−/−^ mice, and fed a HF diet for 10 weeks. Importantly, no difference in body weights (*Figure 2A*) or lipid levels (*Figure 2B*) was found during fat feeding. In addition, no difference in lipid level was seen between *Il1a*^−/−^/*Apoe*^−/−^ and *Apoe*^−/−^ mice (see Supplementary material online, *Figure S1*). Furthermore, no difference in haematological parameters (*Figure 2C*), blood monocyte activation (see Supplementary material online, *Figure S2A*), or activation of the adaptive immune system (see Supplementary material online, *Figure S2B–D*) was seen between groups. Notably, thrombin activity was indistinguishable between groups (see Supplementary material online, *Figure S3*). Together this again supports that mutation of the thrombin site in IL-1α does not change systemic parameters that are known to alter atherosclerosis. However, after 10 weeks of HF diet, aortic root plaques were significantly smaller in IL-1αTM/*Apoe*^−/−^ mice compared with *Apoe*^−/−^ mice, with the same finding in two separate experiments analysed by either ‘traditional’ peak plaque measurement (*Figure 2D* and *E*) or importantly plaque serial sectioning (*Figure 2F*), with significantly smaller area under the curve (AUC; *Figure 2G*), peak root plaque (*Figure 2H*), and single largest plaque (*Figure 2I*) in IL-1αTM/*Apoe*^−/−^ mice. Interestingly, no difference in % plaque coverage of the aorta was seen following Oil red O staining (see Supplementary material online, *Figure S4*), perhaps because the haemodynamic forces within the aorta do not favour the plaque erosion/rupture needed to activate thrombin and enable IL-1α activation. Together, this suggests that thrombin-activated IL-1α normally contributes to plaque growth.

### 3.3 Plaque composition is altered in IL-1αTM/*Apoe*^−/−^ mice

**Figure 3:**
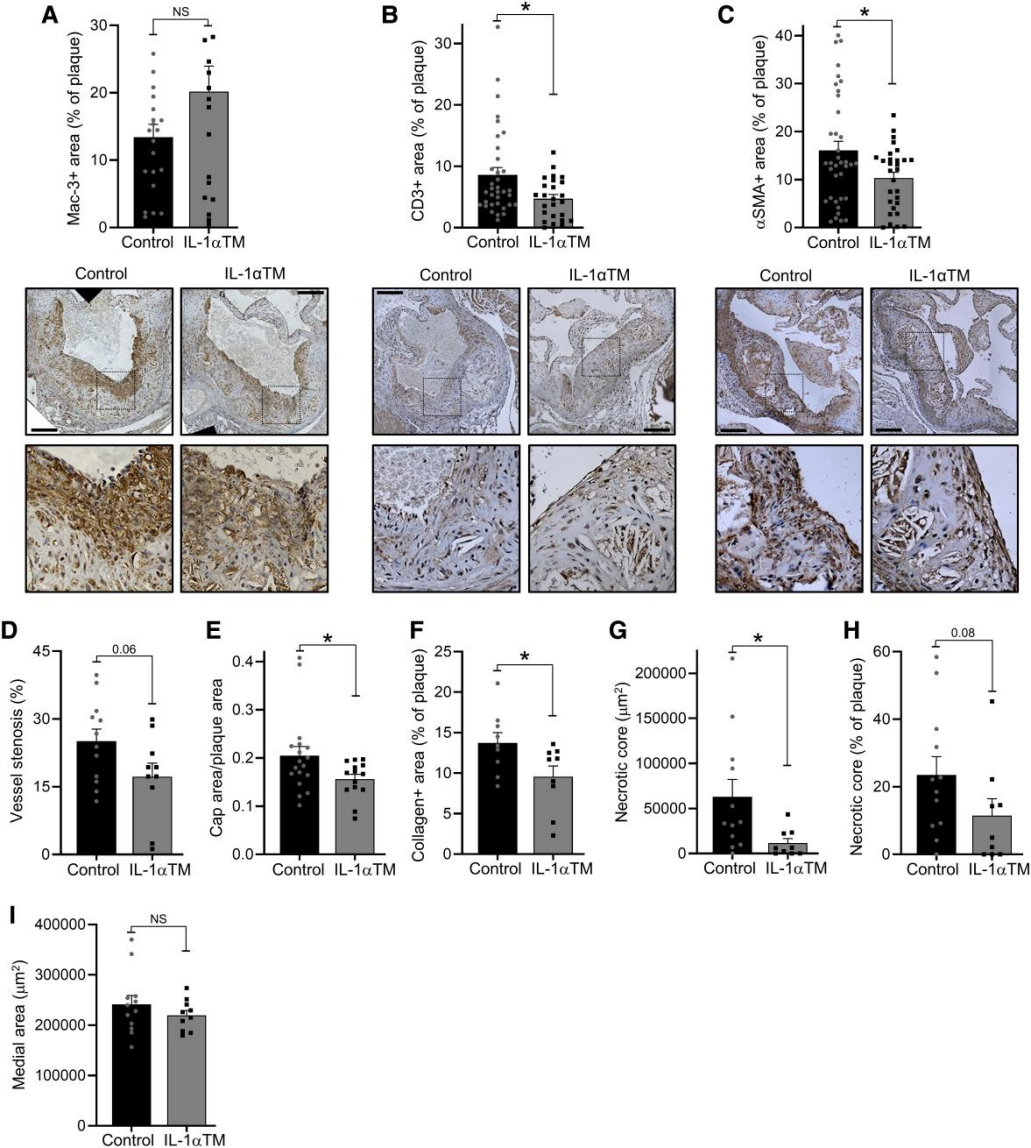
Plaque composition is altered in IL-1αTM/*Apoe*^−/−^ mice. (*A–C*) *Apoe*^−/−^ (control) and IL-1αTM/*Apoe*^−/−^ (IL-1αTM) mice were fed an HF diet for 10 weeks, aortic root plaques stained for Mac-3 (*A*), CD3 (*B*), and αSMA (*C*), and % of the plaque staining for the marker enumerated, with representative images below, and boxed sections magnified. (*D–I*) Aortic roots were also analysed for the level of vessel stenosis (*D*), the ratio of fibrous cap area to plaque area (*E*), collagen content (*F*), necrotic core total area (*G*), necrotic core as a % of plaque (*H*), and vessel medial area (*I*). Data represent mean ± SEM; *n* = 12/10 (control/IL-1αTM) mice, or number of individual plaques as indicated. *P = ≤ 0.05, **P ≤ 0.01; NS = not significant. Scale bar = 200 μm.

Although plaque size affects vessel stenosis, plaque composition dictates stability and thus probability of acute rupture. No significant difference in the number of plaque Mac-3^+^ cells (typically indicative of macrophages; *Figure 3A*) was found, but significantly less CD3^+^ cells (typically indicative of T cells; *Figure 3B*) and αSMA^+^ cells (typically indicative of VSMCs; *Figure 3C*) were seen in IL-1αTM/*Apoe*^−/−^ lesions (see Supplementary material online, *Figure S5*for IHC controls). Furthermore, CD8 T cells from IL-1αTM/*Apoe*^−/−^ mice polarized less towards IFNγ expression after *in vitro* stimulation (see Supplementary material online, *Figure S2E*). More inflammatory cells and less VSMCs typically indicate a more unstable plaque. Thus, less thrombin-activated IL-1α in IL-1αTM mice not only lowers CD3^+^ cell recruitment, but also reduces VSMC content—perhaps indicating that although IL-1 signalling drives atherogenesis, it may also have a stabilizing effect via promotion of VSMC proliferation. Indeed, although the smaller plaques in IL-1αTM/*Apoe*^−/−^ mice resulted in less vessel stenosis (*Figure 3D*), they also have smaller fibrous caps relative to plaque area (*Figure 3E*) and reduced collagen content (*Figure 3F*), in keeping with the reduced VSMC content witnessed (*Figure 3C*), and indicative of a more unstable plaque phenotype. Plaques in IL-1αTM/*Apoe*^−/−^ mice also have smaller necrotic cores (*Figure 3G* and *H*), but medial area was the same between groups (*Figure 3I*), indicating no alteration to vessel wall remodelling (see Supplementary material online, *Figure S6*, for example morphometry). Together, this indicates that thrombin-cleaved IL-1α normally drives atherogenesis and recruitment and/or retention of CD3^+^ immune cells, but with a potentially unexpected role in plaque stabilization via increased VSMC and collagen content.

### 3.4 Thrombin inhibition does not reduce atherogenesis in IL-1αTM/*Apoe*^−/−^ mice

**Figure 4:**
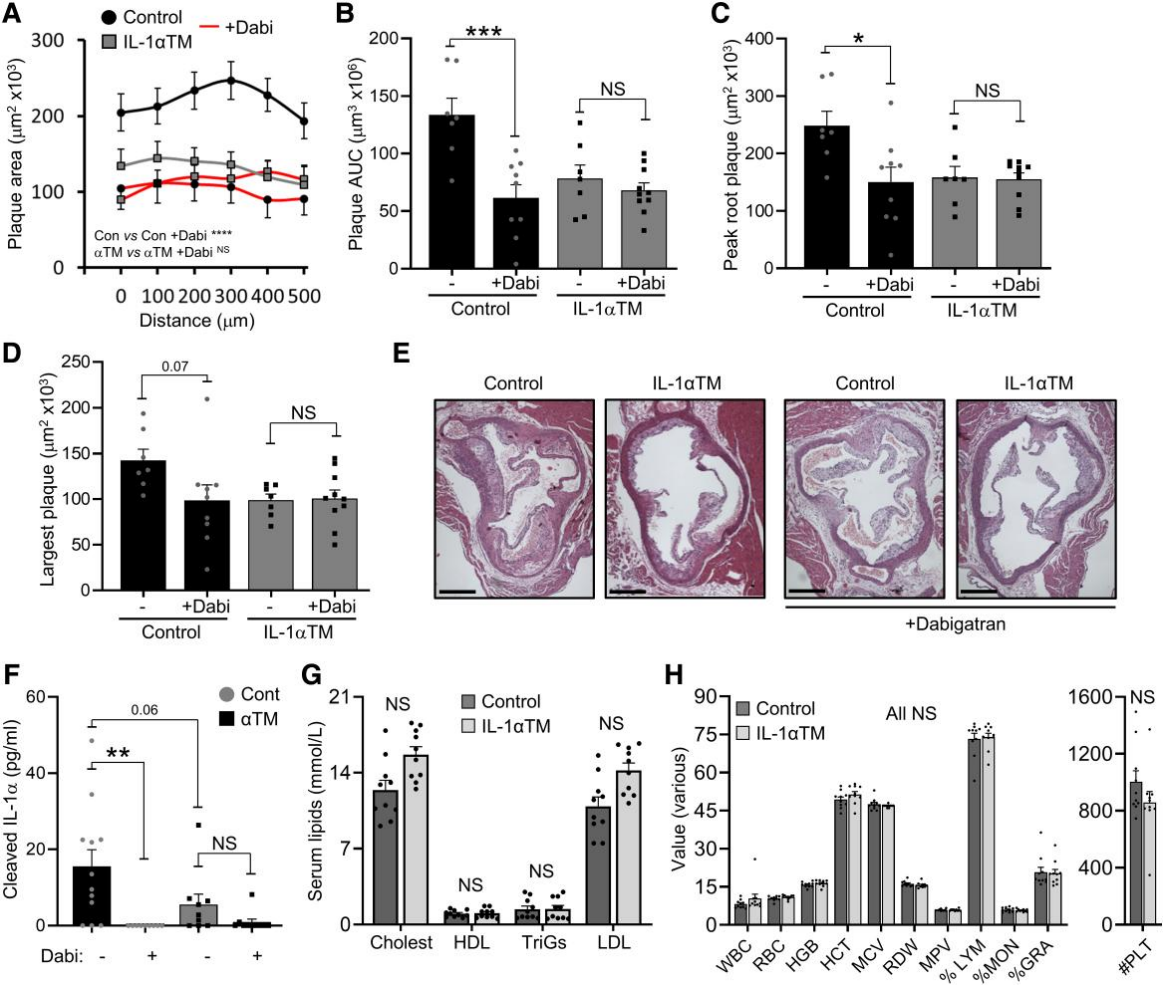
Thrombin inhibition does not reduce atherogenesis in IL-1αTM/*Apoe*^−/−^ mice. *Apoe*^−/−^ (control) and IL-1αTM/*Apoe*^−/−^ (IL-1αTM) mice were fed an HF diet for 10 weeks, ± the thrombin inhibitor Dabigatran (+Dabi), with aortic root plaque serial sectioned, quantified (*A*) and analysed for area under the curve (*B*), peak plaque area (*C*), and single largest plaque (*D*), along with representative images (*E*). (*F*) Serum-cleaved IL-1α level by ELISA at 10 weeks in mice treated ±Dabigatran. (*G* and *H*) Lipid concentrations (*G*) and full blood counts (*H*) measured at 6 weeks in mice receiving dabigatran. Data represent mean ± SEM; *n* = 7/9/7/10 (*A–D*), 10/10 (*G*, *H*), 12/9/9/10 (*F*). *P = ≤ 0.05, **P ≤ 0.01, ***P ≤ 0.001, ****P ≤ 0.0001; NS = not significant. Scale bar = 500 mm.

Acute plaque rupture causes thrombosis, vessel occlusion, and MI/stroke, while ongoing activation of coagulation drives plaque growth.^5–9^ Indeed, thrombin inhibition is shown to reduce atherosclerosis by approximately half,^10,11^ but it is not known if this action could be, in part, via reduced thrombin activation of IL-1α. To investigate this, we utilized the oral anticoagulant Dabigatran to inhibit thrombin activity during atherogenesis and compared plaque size and serum IL-1α level between IL-1αTM/*Apoe*^−/−^ and *Apoe*^−/−^ mice. Thrombin activity was reduced by Dabigatran treatment via diet, as evidenced by reduced TAT complexes in serum (see Supplementary material online, *Figure S7*). Interestingly, although Dabigatran reduced plaque AUC, peak plaque, or largest plaque size by ∼60% in *Apoe*^−/−^ mice (*Figure 4A–E*), a finding in keeping with previous studies,^10,11^ no significant decrease in plaque size was seen in IL-1αTM/*Apoe*^−/−^ mice (*Figure 4A–E*). Importantly, Dabigatran treatment also significantly reduced serum IL-1α levels in *Apoe*^−/−^, but not IL-1αTM/*Apoe*^−/−^ mice, which already had lower serum IL-1α levels without Dabigatran (*Figure 4F*). Importantly, Dabigatran treatment did not alter serum lipid levels (*Figure 4G*) or haematological parameters (*Figure 4H*) between groups. Together, these data suggest that a new mode of action for thrombin inhibitors that retard atherogenesis is, in part, by preventing IL-1α cleavage and activation by thrombin.

### 3.5 Atherogenesis is driven by thrombin-cleaved IL-1α derived from vessel wall and myeloid cells

**Figure 5:**
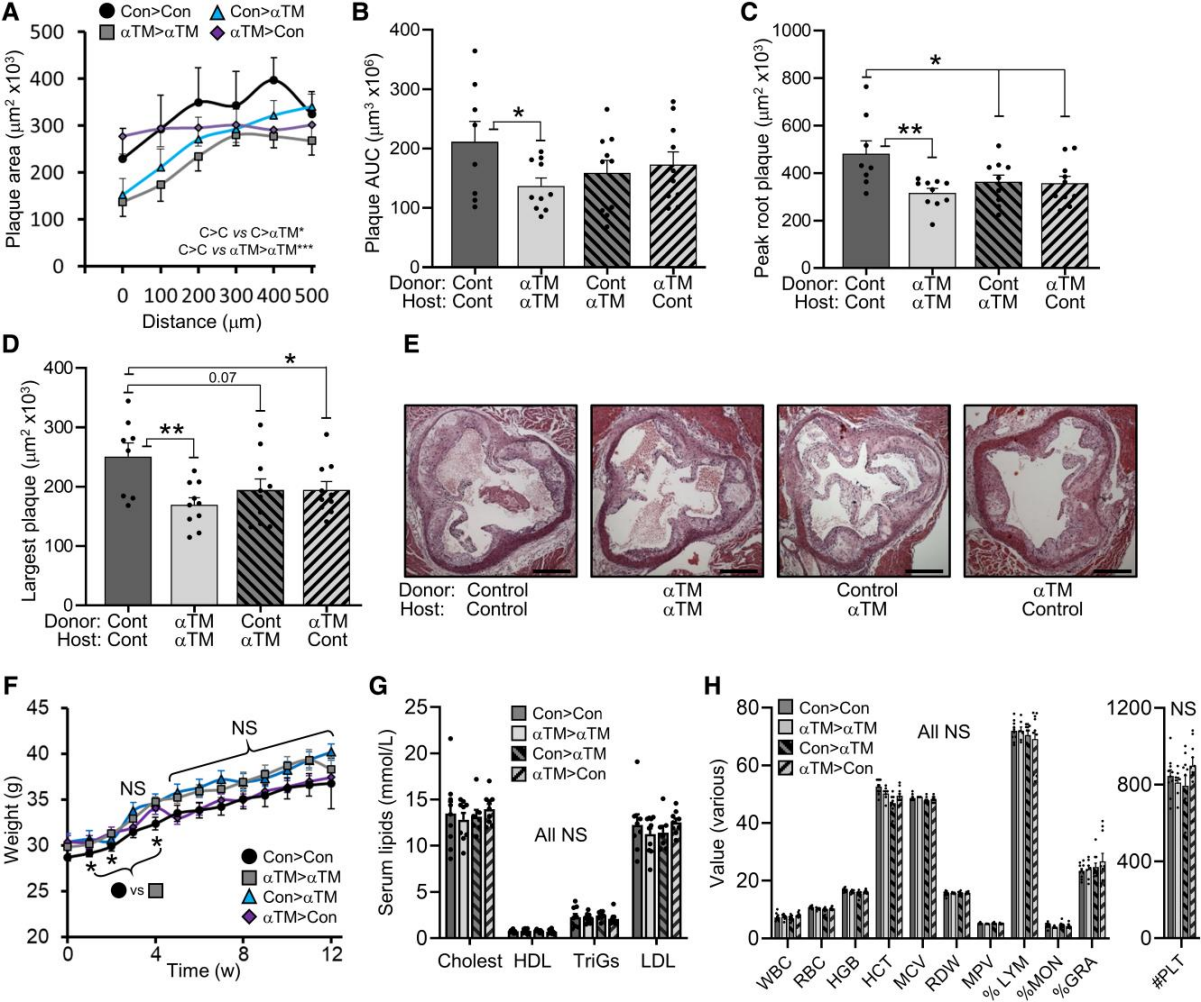
Atherogenesis is driven by thrombin-cleaved IL-1a derived from vessel wall and myeloid cells. *Apoe*^−/−^ (control) and IL-1αTM/*Apoe*^−/−^ (IL-1αTM) syngeneic and congenic bone marrow chimeras, as indicated, were fed an HF diet for 12 weeks and aortic root plaque serial sectioned, quantified (*A*) and analysed for area under the curve (*B*), peak plaque area (*C*), and single largest plaque (*D*), along with representative images (*E*). (*F–H*) Longitudinal body weight (*F*) and serum lipid concentrations (*G*) and full blood counts (*H*) measured at 6 weeks of fat feeding. Data represent mean ± SEM; *n* = 8/10/10/10 (*A–D*), 9/10/10/10 (*F–H*). *P = ≤ 0.05, **P ≤ 0.01, ***P ≤ 0.001; NS = not significant.

pro-IL-1α is expressed constitutively by most non-myeloid cells,^3^ including VSMCs^25^ and ECs,^42^ while myeloid expression requires stimulation by, for example, TLR ligands. To investigate the source of the pro-IL-1α activated by thrombin during atherogenesis, we generated congenic bone marrow chimeras (IL-1αTM/*Apoe*^−/−^ > *Apoe*^−/−^ and *Apoe*^−/−^ > IL-1αTM/*Apoe*^−/−^), along with syngeneic controls (IL-1αTM/*Apoe*^−/−^ > IL-1αTM/*Apoe*^−/−^ and *Apoe*^−/−^ > *Apoe*^−/−^). Engraftment level was ∼95% for both *Apoe*^−/−^ and IL-1αTM/*Apoe*^−/−^ bone marrow (see Supplementary material online, *Figure S8A* and *B*), and engraftment rate was equivalent between all groups (see Supplementary material online, *Figure S8C*). Plaque AUC, peak plaque, and largest plaque remained smaller in syngeneic IL-1αTM/*Apoe*^−/−^ transplanted mice compared with syngeneic *Apoe*^−/−^ (*Figure 5A–E*), in keeping with our previous data without bone marrow transplant (*Figure 2D–I*). Interestingly, reduced plaque size was seen in both congenic IL-1αTM/*Apoe*^−/−^ > *Apoe*^−/−^ and *Apoe*^−/−^ > IL-1αTM/*Apoe*^−/−^ transplants (*Figure 5A–E*), indicating thrombin-activated pro-IL-1α can be derived from both vessel wall and myeloid cells during atherogenesis. Importantly, no difference in body weight (*Figure 5F*), serum lipid levels (*Figure 5G*), or haematological parameters (*Figure 5H*) was seen between groups, excluding an effect of genotype on bone marrow repopulation or, for example, leucocyte levels. Together, this again supports that thrombin-cleaved pro-IL-1α promotes atherogenesis, but that the source of pro-IL-1α is not critical.

### 3.6 IL-1-driven nuclear factor–κΒ activation increases VSMC proliferation

**Figure 6:**
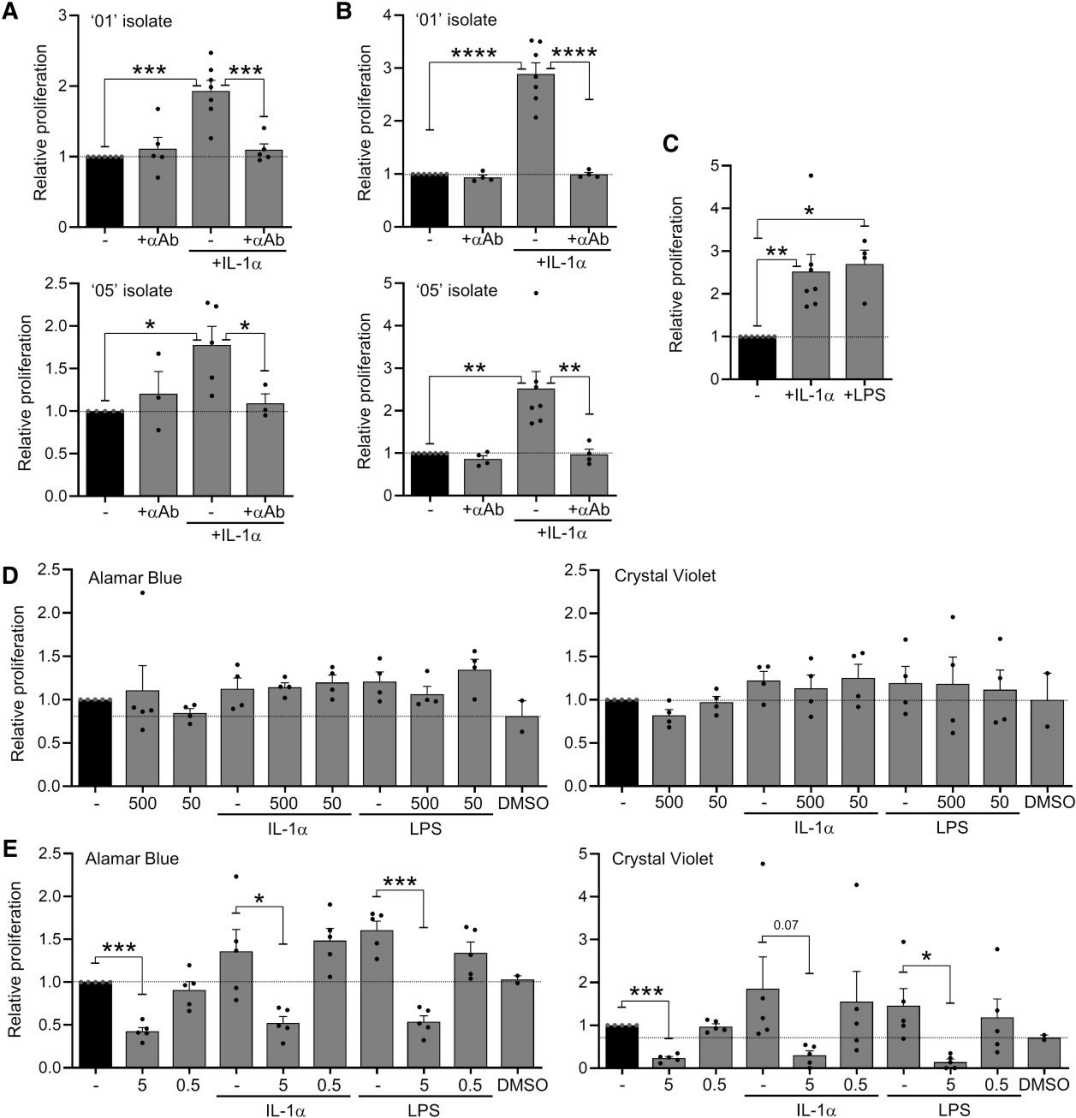
IL-1-driven NF-κB activation increases VSMC proliferation. (*A* and *B*) Relative proliferation rate of different ‘01’ or ‘05’ isolates of human primary VSMCs treated ± IL-1α, ± an IL-1α neutralizing antibody (+αAb), as measured with Alamar Blue (*A*) or Crystal Violet (*B*). (*C*) Relative proliferation rate of ‘05’ VSMCs treated ±IL-1α or ±LPS, as measured with Crystal Violet. (*D* and *E*) Relative proliferation rate of VSMCs treated ±IL-1α or ±LPS, ± the p38 MAP kinase inhibitor SB203580 (500/50 nM) (*D*), or ± the NF-κB inhibitor Isohelenin (5/0.5 mM) (*E*), measured with Alamar Blue or Crystal Violet, as indicated. Dotted line indicates the lower value of untreated VSMCs or DMSO control. Data represent mean ± SEM; *n* = 3–7 (*A*), 4–7 (*B*, *C*), 4 (*D*), 5 (*E*); *P = ≤ 0.05, **P ≤ 0.01, ***P ≤ 0.001, ****P ≤ 0.0001; NS = not significant.

Plaques with fewer VSMCs, less collagen and smaller fibrous caps in IL-1αTM/*Apoe*^−/−^ mice (*Figure 3*C, E, and *F*) suggests that in addition to driving atherogenesis, IL-1α may also play a positive role via plaque stabilization. To investigate this, we cultured two different primary VSMC isolates with IL-1α for 7 days and measured proliferation with Alamar Blue. IL-1α resulted in a significant increase in Alamar Blue signal, which was entirely reversed with an IL-1α neutralizing antibody (*Figure 6A*). However, because Alamar Blue can sometimes be confounded by increased metabolic activity, we repeated experiments but measured VSMC proliferation by cell mass with crystal violet, which essentially gave the same results (*Figure 6B*). As IL-1α signalling via IL-1R1 utilizes MyD88, akin to TLR4, we also treated VSMCs with LPS, which also increased proliferation (*Figure 6C*). IL-1 and LPS signalling downstream of MyD88 can engage mitogen-activated protein kinases and nuclear factor-κB (NF-κB) activation. Thus, to determine the pathway-inducing VSMC proliferation, we utilized the p38 MAP kinase inhibitor SB203580 and the NF-κB inhibitor isohelenin. SB203580 had no effect on VSMC proliferation over and above any effect of the DMSO carrier control (*Figure 6D*). However, isohelenin reduced both basal VSMC proliferation and that induced by IL-1α or LPS (*Figure 6E*). Finally, we measured expression of the vascular collagen genes *COL1A1*, *COL1A2*, and *COL3A1* in VSMCs treated ± IL-1α, which revealed a modest increase in expression (see Supplementary material online, *Figure S9*), in keeping with previous findings.^43^ Together, this suggests that in addition to an atherogenic role for IL-1,^26–36^ IL-1-induced NF-κB activation may help stabilize plaques via directly increasing VSMC number, along with their associated production of structural matrix.

## 4. Discussion

With the transition to larger multicellular organisms came, the need to keep blood and nutrients in and pathogens out, and thus the evolution of a rudimentary combined immuno-coagulation system. Indeed, simple organisms with combined coagulation and immune systems are still extant today, underscoring the importance of coordinating these processes. Although modern mammals have evolved increasingly complex immune and coagulation systems, essential links between them still exist, including the activation of IL-1α by thrombin.^4^ Atherosclerosis is driven by chronic aberrant immune activation, with the major clinical outcome driven by acute occlusive thrombosis. However, the effect of long-term crosstalk between coagulation and immunity on atherogenesis is still poorly understood.

We have studied one aspect of this interplay using a novel mouse model in which endogenous IL-1α has been mutated to prevent its activation by thrombin. We find that IL-1αTM/*Apoe*^−/−^ mice generate smaller atherosclerotic plaques after fat feeding, but with no change to systemic parameters such as lipids or leucocyte counts. IL-1αTM/*Apoe*^−/−^ plaques have fewer T cells, but also less VSMCs, collagen and smaller fibrous caps, suggesting that although thrombin-activated IL-1α can drive atherogenesis, it can also induce features associated with plaque stabilization. Indeed, we show that IL-1α potently drives VSMC proliferation via NF-κB. Interestingly, the well-reported reduction in plaque size upon thrombin inhibition was absent in IL-1αTM/*Apoe*^−/−^ mice, suggesting the action of thrombin inhibitors on atherosclerosis are, in part, via reduced IL-1α activation. Finally, bone marrow chimeras reveal that pro-IL-1α cleaved by thrombin can be derived from either vessel wall or myeloid cells.

The most pathological role of coagulation in atherosclerosis is the production of vessel-occluding thrombi after plaque rupture. Plaque rupture both interrupts continuity of the endothelial layer and exposes thrombogenic material within the necrotic core, often leading to MI or stroke. However, the systemic response to MI actively accelerates atherogenesis via increased monocyte recruitment,^44^ suggesting downstream effects of acute coagulation can drive chronic inflammation. IL-1 is a powerful inducer of endothelial adhesion molecule expression that leads to monocyte recruitment, but if thrombin-activated IL-1α drives this is currently unknown. Similarly, repeated rounds of subclinical plaque rupture and erosion, followed by repair, are also suggested to drive atherogenesis.^45^ Indeed, layers of fibrin deposition witnessed throughout human plaques support this,^5^ and could explain how chronic low-grade coagulation provides thrombin to activate local IL-1α and drive plaque growth. Much of the crosstalk between coagulation and immunity is via thrombin activation of PARs, which instigate cell signalling via G-proteins to ultimately induce proinflammatory cytokines. However, despite the large effect of thrombin inhibitors on plaque growth, PAR-deficient mice either show a modest reduction^13^ or no change^12^ in plaque size, suggesting that thrombin influences atherogenesis via other mechanisms. Our data indicate that cleavage and activation of IL-1α may in part explain some of the PAR-independent effects of thrombin on atherosclerosis.

Notably, we did not see altered plaque coverage of the aorta between groups. However, serial sectioning of aortic roots is a proxy for plaque volume (i.e. μm3), while staining aortas en face measures plaque area (i.e. μm2). Thus, processes initiating plaque formation that alter % coverage are likely different to processes that drive subsequent plaque growth to alter volume. Given that thrombin cleavage of IL-1α would likely need an established plaque environment to occur, it is plausible that thrombin/IL-1α does not initiate athero formation, but rather accelerates plaque growth. Alternatively, haemodynamic forces within the aorta may not favour the plaque erosion/rupture that is needed to activate thrombin and enable IL-1α activation.

In addition to smaller plaques, lesion composition in IL-1αTM/*Apoe*^−/−^ mice was also altered. Significantly reduced numbers of CD3^+^ T cells were found, suggesting that IL-1α normally recruits and/or retains T cells in the plaque. In addition, spleen CD8^+^ T cells from IL-1αTM/*Apoe*^−/−^ mice also produce significantly less IFNγ, perhaps indicative of less interaction with antigen-presenting cells. Interestingly, IFNγ-producing CD8^+^ T cells have been shown to be persistently higher in patients with coronary artery disease with acute coronary syndrome and stable angina,^46^ with human plaques showing more activated CD8 than CD4.^47^ Indeed, IL-1 induces expression of multiple CXC chemokines able to recruit CXCR3^+^ T cells.^48^ Lesions in IL-1αTM/*Apoe*^−/−^ mice also have less VSMCs and collagen and relatively smaller fibrous caps, which are related given that VSMCs produce the collagen that supports fibrous caps. This hints at a dual role for IL-1 signalling in driving both atherogenesis and plaque stabilization. Indeed a report with global *Il1r1* knockout resulted in smaller plaques that were unstable,^38^ while IL-1β neutralization or *Il1r1* deletion in VSMCs reduces VSMC number and fibrous cap size.^49^ We also show that IL-1 not only strongly promotes proliferation of VSMCs via NF-κB, supporting previous work,^50^ but also it upregulates VSMC collagen expression. Thus, we have the paradox where IL-1 promotes VSMC-mediated plaque stabilization in mice, but IL-1β blockade lowers MACE^14^ in humans that are typically caused by rupture of unstable plaques.

Thrombin inhibitors are reported to reduce atherosclerosis in mice,^10,11^ but whether this is via reduced coagulation or inflammation is not clear. Much of the action of thrombin on inflammation is thought to be via PARs, but as stated above, PAR2 or PAR4 loss has no or minimal effect on plaque size.^12,13^ While some mouse atherosclerosis studies show reduced inflammatory markers with thrombin inhibition,^11,51^ treatment of post-MI patients with the direct thrombin inhibitor Ximelagatran increased serum IL-18 and CRP levels.^52^ In addition, a thrombin-driven factor XI feedback loop on platelets drives vascular inflammation and hypertension, which could also involve IL-1α activation.^53^ In terms of cardiovascular risk, HORIZONS-AMI indicated thrombin inhibition with bivalirudin after STEMI did not alter MACE but did lower mortality,^54^ albeit to a small degree, while the LURIC study reported an inverse correlation between cardiovascular risk and endogenous thrombin potential, suggesting a low level of thrombin activity may actually be protective.^55^ Clearly acute coagulation and the downstream inflammatory sequelae of ischaemia have profound effects on patients with cardiovascular disease, but how ongoing coagulation and thrombin activity affect atherogenesis and plaque stability is still unknown. Our finding that IL-1αTM/*Apoe*^−/−^ mice are refractory to the reduction in plaque size seen with thrombin inhibitors suggests a new mode of action for thrombin inhibitors, in part via the reduced production of active IL-1α. However, whether this new link drives cardiovascular risk and/or mortality in patients is currently unknown.

Finally, percutaneous coronary angioplasty (i.e. stenting) is a common intervention that widens the vessel lumen and increases blood flow. Stent implantation causes significant damage to the endothelium that results in acute thrombus formation that often resolves itself, although impaired re-endothelialization can cause subsequent stent thrombosis. However, in many cases, VSMC proliferation around the stent results in re-narrowing of the vessel in a classic ‘response to injury’ reaction occurring ∼3–12 months after stenting. We have previously shown that damaged VSMCs and ECs release IL-1α,^25,42^ and that thrombin is a potent activator of IL-1α that can be presented on activated platelets.^4^ Here, we show that IL-1α is a potent inducer of VSMC proliferation and collagen. Interestingly, polymorphisms in the IL-1RA gene that increase its plasma concentration, and therefore, reduce IL-1 activity, are significantly associated with reduced restenosis.^56,57^ Thus, the potential exists for the immediate damage caused during stenting and subsequent thrombus formation to be a source of IL-1α that acts on VSMCs to induce their proliferation and cause restenosis.

In conclusion, we show that IL-1α activation by thrombin represents a previously unappreciated point of interplay between coagulation and immunity that promotes atherosclerosis, and that the inhibitory action of thrombin inhibitors on plaque growth are mediated, in part, via thrombin activation of IL-1α. However, IL-1α may also play an important role in stabilizing lesions via induction of VSMC proliferation and collagen, and thus therapeutic targeting of IL-1 and/or thrombin clearly requires more research.

## Supplementary Material

cvad091_Supplementary_Data

## Data Availability

The data supporting this study are available from the corresponding author under resonable request.

## Notes

Translational perspective Inflammation drives atherogenesis and plaque rupture, while coronary-occluding thrombi cause myocardial infarction. However, coagulation and immunity are linked by thrombin activation of IL-1α. Using a mutant mouse in which thrombin cannot activate IL-1α shows smaller atherosclerotic plaques with reduced VSMCs, collagen and fibrous caps, indicating an unstable plaque phenotype. Importantly, thrombin inhibitors that normally retard atherogenesis don't affect plaques in mutant mice, suggesting they alter atherosclerosis via reducing IL-1α activation. Thus, coagulation can drive atherogenesis, in part, via thrombin cleavage of IL-1α. This research highlights the potential for therapeutically targeting IL-1α/thrombin, but also forewarns that IL-1 may stabilise atherosclerotic plaques.
