# Erratum to: Thrombin-activated interleukin-1α drives atherogenesis, but also promotes vascular smooth muscle cell proliferation and collagen production

**DOI:** 10.1093/cvr/cvad128

**Published:** 2023-08-22

This is an erratum to: Laura C Burzynski and others, Thrombin-activated interleukin-1α drives atherogenesis, but also promotes vascular smooth muscle cell proliferation and collagen production, *Cardiovascular Research*, Volume 119, Issue 12, August 2023, Pages 2179–2189, https://doi.org/10.1093/cvr/cvad091

In the originally published version of this manuscript, “NF-kB” was incorrectly expanded to “necrosis factor kB” instead of “nuclear factor kB”. This error was present in the title and text of section 3.6.

This error, for which the publisher apologizes, has been corrected.

