# Supplementary material for: Thrombin-activated interleukin-1α drives atherogenesis, but also promotes vascular smooth muscle cell proliferation and collagen production
Source: Cardiovasc Res. 2023 Jun 13;119(12):2179–89. doi: 10.1093/cvr/cvad091 (PMC10578913; doi:10.1093/cvr/cvad091)
Supplement: cvad091_Supplementary_Data [file cvad091_supplementary_data.zip › IL-1aTM AtheroPaper Supplementary Material.docx]

Thrombin activated Interleukin-1α drives atherogenesis, but also promotes VSMC proliferation and collagen production

Laura C. Burzynski^1^, Maria A. Morales-Maldonado^1^, Amanda Rodgers^1^, Lauren A. Kitt^1^, Melanie Humphry^1^, Nichola Figg^1^, Martin R. Bennett^1^, Murray CH. Clarke^1^*

*** For correspondence**

E-Mail: [mchc2@cam.ac.uk](mailto:mchc2@cam.ac.uk)

Telephone: (44) 1223 762581

Fax: (44) 1223 331505

^1^Section of CardioRespiratory Medicine,

The Heart & Lung Research Institute,

The University of Cambridge,

Papworth Road,

Cambridge Biomedical Campus,

Cambridge,

CB2 0BB,

UK.

**Running title:** Thrombin activated IL-1α drives atherogenesis.

**Keywords:** Atherosclerosis, inflammation, coagulation, IL-1, thrombin.

Supplementary Material

**SUPPLEMENTARY FIGURE LEGENDS**

**Figure S1: Serum lipids are equivalent between *Apoe*^-/-^ *Il1a*^-/-^/*Apoe*^-/-^ mice.** *Apoe*^-/-^ and *Il1a*^-/-^/*Apoe*^-/-^ mice were fed a high fat diet for 6 w and serum lipids measured. Data represent mean ± SEM; n = 10/10; NS = not significant.

**Figure S2: CD8 T cells in fat fed IL-1αTM/*Apoe*^-/-^ mice produce less IFNγ.** *Apoe*^-/-^ (control) and IL-1αTM/*Apoe*^-/-^ (IL-1αTM) mice were fed a high fat diet for 10 w before assessment of circulating myeloid cells **(A)**, splenic CD4/8 T cell level **(B)** and subtype **(C)**, splenic Treg level **(D)**, and splenic CD4/8 T cell polarisation after PMA/Ionomycin **(E)**. Data represent mean ± SEM; n = 4/4 (A), 7/7 (B-E). p = *≤0.05; NS = not significant.

**Figure S3: Thrombin activity is equivalent between *Apoe*^-/-^ and IL-1αTM/*Apoe*^-/-^ mice.** *Apoe*^-/-^ (Control) and IL-1αTM/*Apoe*^-/-^ (IL-1αTM) mice were fed a high fat diet for 6 w and serum thrombin activity measured with a fluorogenic substrate assay. Dil = serum dilution. Data represent mean ± SEM; n = 3; NS = not significant.

**Figure S4: Plaque coverage of the aorta is not different between *Apoe*^-/-^ and IL-1αTM/*Apoe*^-/-^ mice. (A,B)** *Apoe*^-/-^ (Control) and IL-1αTM/*Apoe*^-/-^ (IL-1αTM) mice were fed a high fat diet for 10 w, aortas stained with Oil red O to visualise plaque **(A)**, and the area quantified **(B)**. Data represent mean ± SEM; n = 12/10; NS = not significant.

**Figure S5: Mac-3, CD3 and** α**SMA IHC shows specific staining in control tissues.** Sectioned formalin fixed paraffin embedded tissue of mouse carcinoma, thymus and heart stained with isotype controls (negative control) or anti-CD3, anti-Mac-3 or anti-αSMA (positive control), followed by identical development, as detailed in the materials and methods.

**Figure S6: Examples of the delineation of plaque features for morphometry measurements.** **(A-D)** Sectioned formalin fixed paraffin embedded aortic root plaque was stained as indicated. **(A)** Plaque area (red), internal (black) and external (white) elastic lamina (IEL/EEL) were masked, with % stenosis calculated as the area of plaque occupying the area inside the IEL, and the medial area representing the area between the EEL and IEL. **(B)** Fibrous cap (red) and plaque (black) were masked, areas calculated and the ratio of cap to plaque calculated. **(C)** Plaque area (black) was masked, pixels within matching the colour range (blue circle) were selected and % of collagen calculated. **(D)** Necrotic cores (red) and plaque (black) were masked, areas calculated and the % of necrotic core within the plaque, or total necrotic core area calculated.

**Figure S7: Dabigatran administered via diet inhibits thrombin.** Thrombin-antithrombin (TAT) complexes by ELISA in the serum of mice before (Pre) or 6 w after Dabigatran administration via diet (+Dabi). Data represent mean ± SEM; n = 2.

**Figure S8: Engraftment level and rate after bone marrow transplant is equivalent between groups. (A,B)** CD45.2 *Apoe*^-/-^ (Cont) or CD45.2 IL-1αTM/*Apoe*^-/-^ (αTM) bone marrow was adoptively transferred into irradiated CD45.1 mice, allowed to reconstitute for 4 w, and the level of circulating CD45.1 and CD45.2 cells enumerated by flow cytometry **(B)**. **(C)** Full blood counts in *Apoe*^-/-^ (Con) and IL-1αTM/*Apoe*^-/-^ (αTM) syngeneic and congenic bone marrow chimeras, as indicated, after 4 w of reconstitution. Data represent mean ±SEM; n = 3 (A), 10,7,10,11 (C), or representative (B); NS = not significant.

**Figure S9: IL-1α upregulates collagen expression in VSMCs.** qPCR data showing level of *COL1A1*, *COL1A2*, *COL3A1* in primary human VSMCs treated with IL-1α for 6 h. Data represent mean ± SEM; n = 2.
