# Supplementary figures and images for: Thrombin-activated interleukin-1α drives atherogenesis, but also promotes vascular smooth muscle cell proliferation and collagen production
Source: Cardiovasc Res. 2023 Jun 13;119(12):2179–89. doi: 10.1093/cvr/cvad091 (PMC10578913; doi:10.1093/cvr/cvad091)

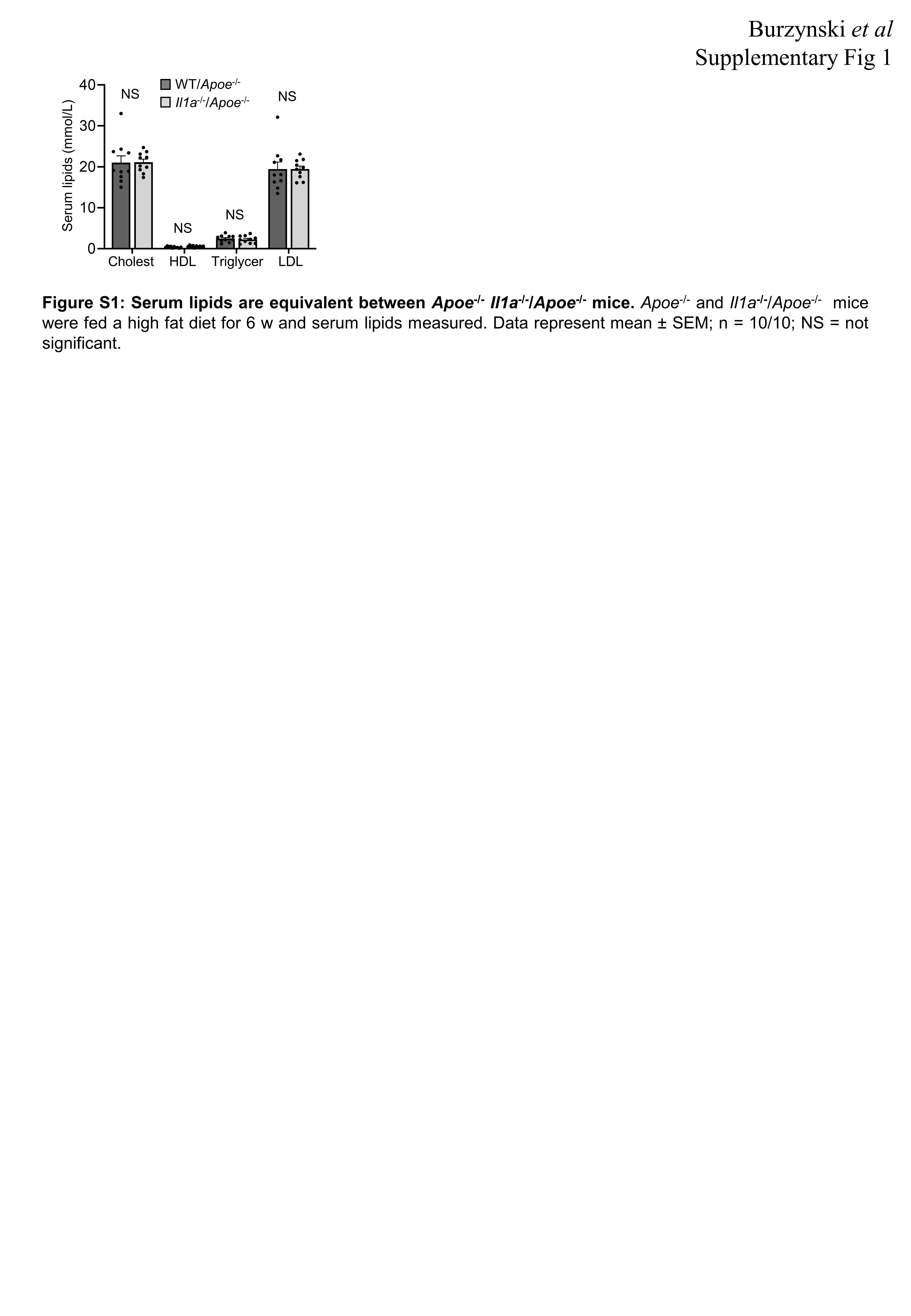

Supplement: cvad091_Supplementary_Data [file cvad091_supplementary_data.zip › Fig S1 R2.jpg]

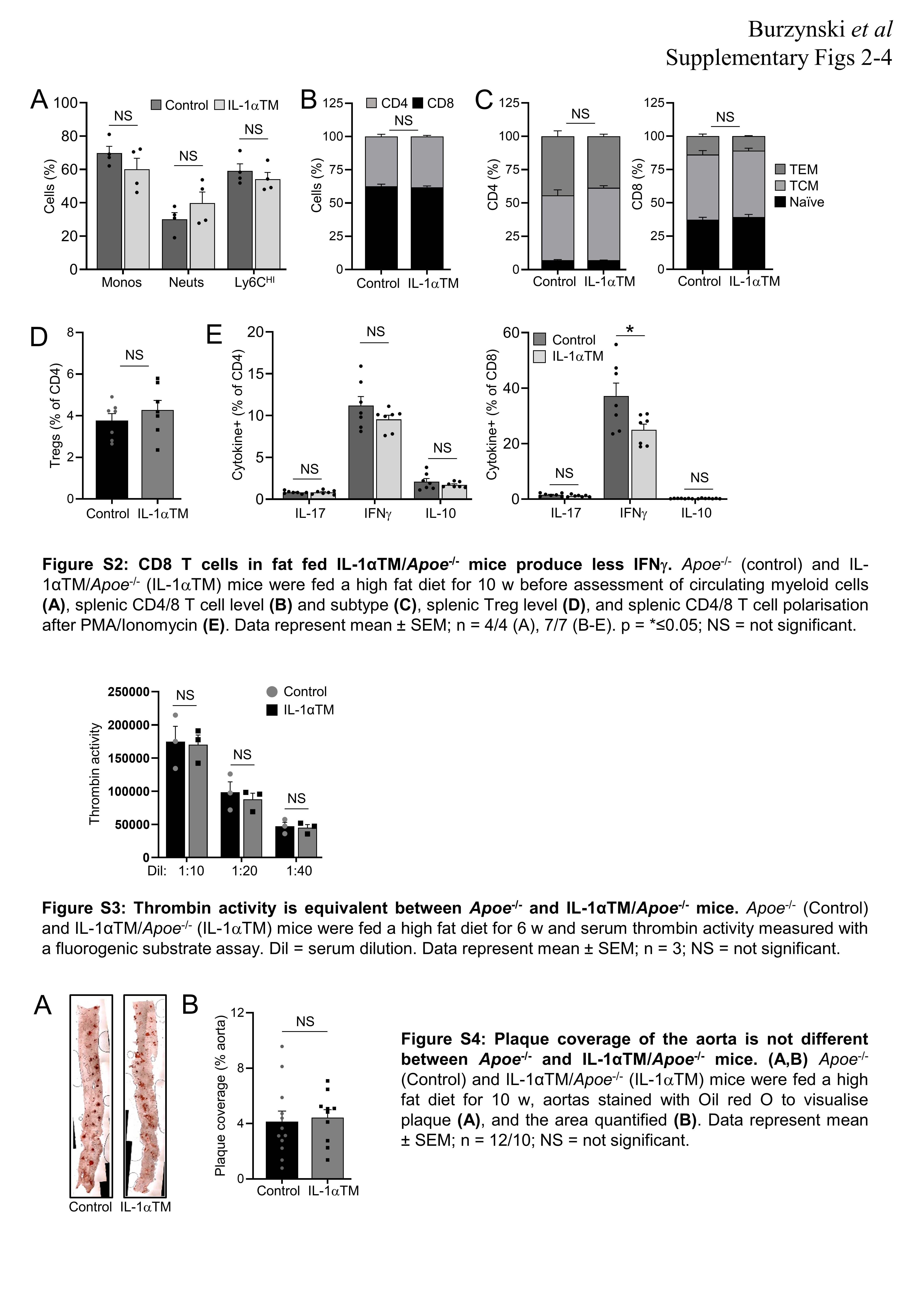

Supplement: cvad091_Supplementary_Data [file cvad091_supplementary_data.zip › Fig S2-4 R2.jpg]

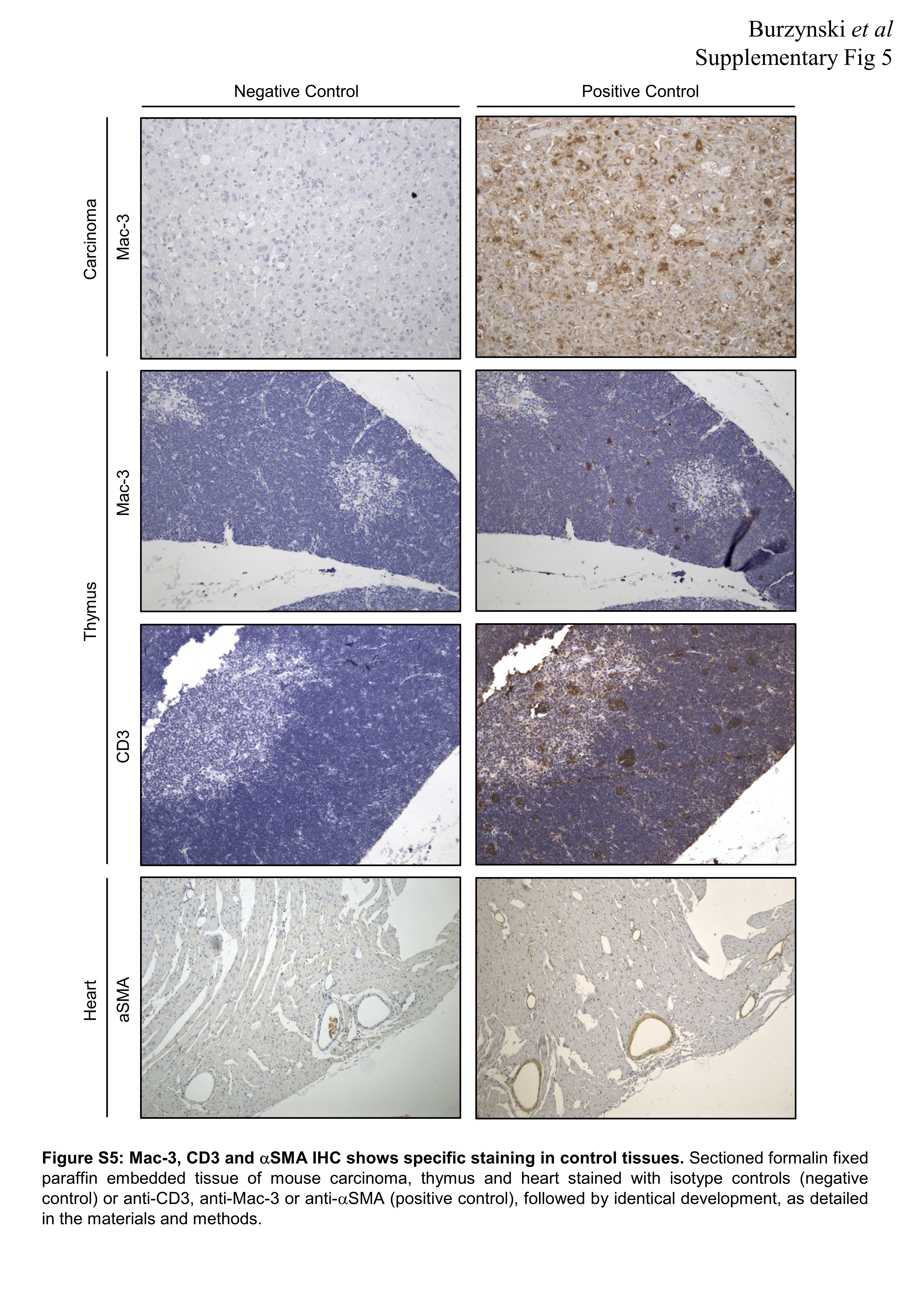

Supplement: cvad091_Supplementary_Data [file cvad091_supplementary_data.zip › Fig S5 R2.jpg]

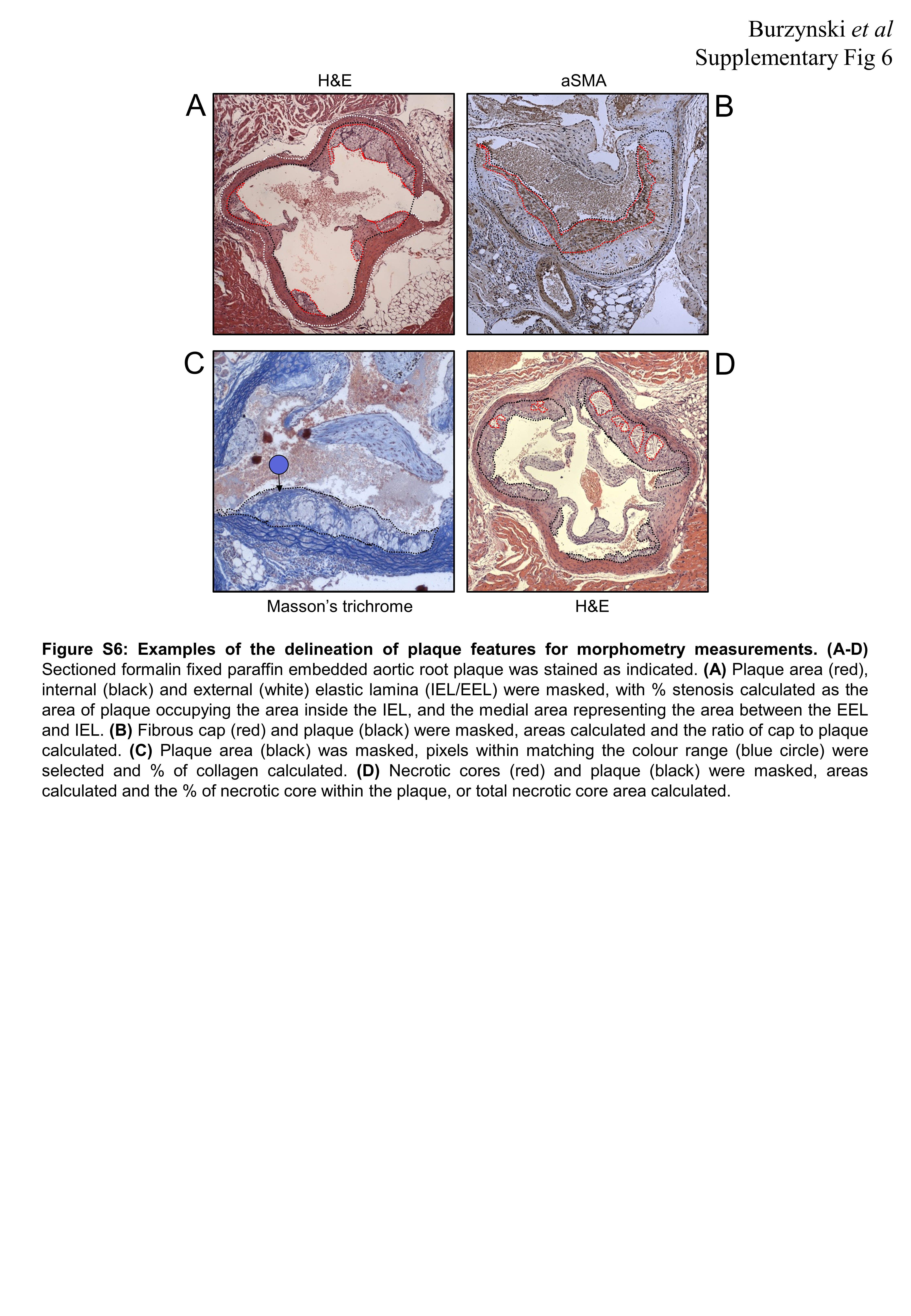

Supplement: cvad091_Supplementary_Data [file cvad091_supplementary_data.zip › Fig S6 R2.jpg]

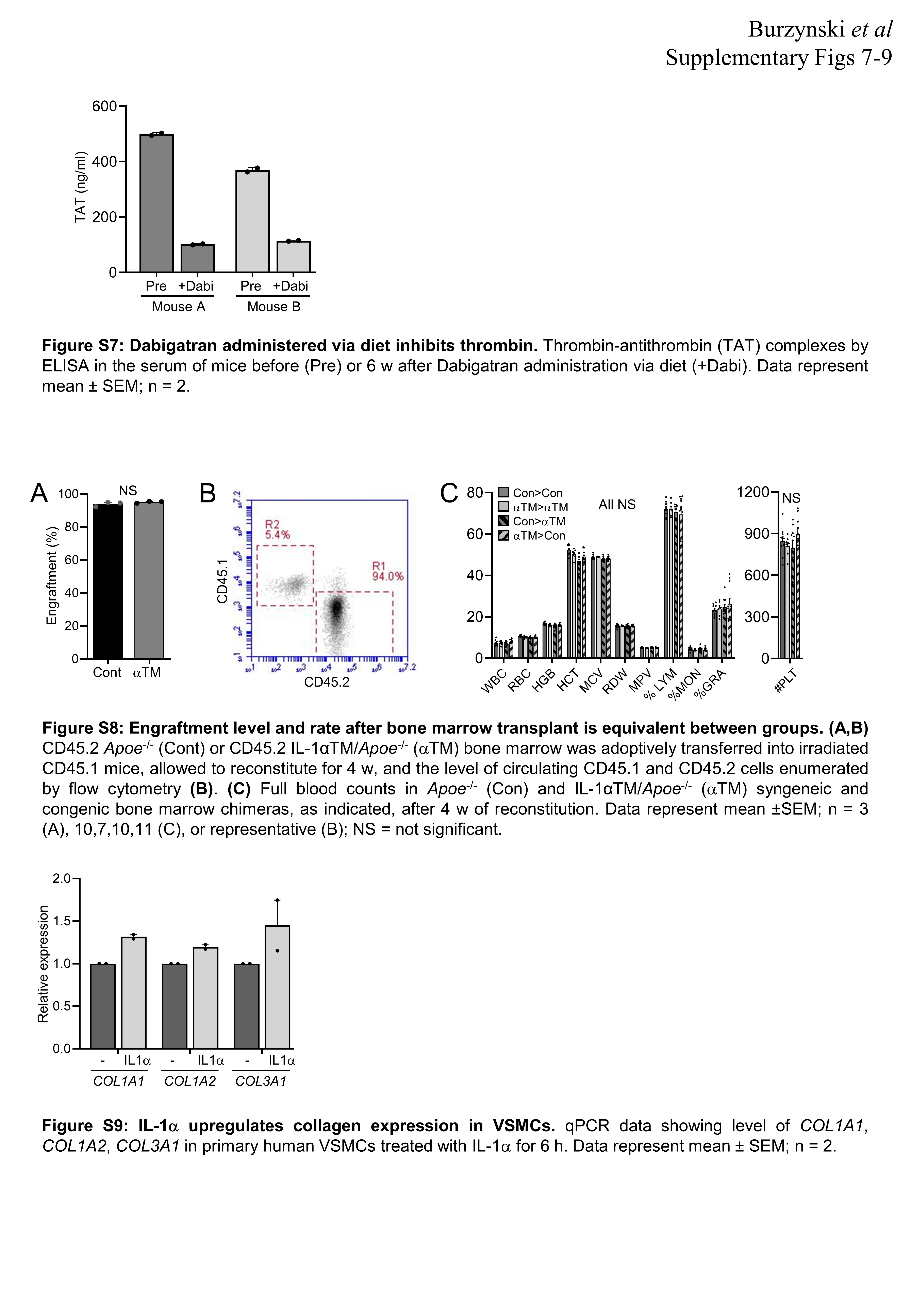

Supplement: cvad091_Supplementary_Data [file cvad091_supplementary_data.zip › Fig S7-9 R2.jpg]
